# Equanimity About Evidence Supporting Early Phase Clinical Trials: A Qualitative Study of Clinician Investigators

**DOI:** 10.21203/rs.3.rs-11167590/v1

**Published:** 2026-10-27

**Authors:** Nicolas Diaz de Leon, Jennifer R. Fishman, Jonathan Kimmelman

**Affiliations:** McGill University

**Keywords:** Neuro-Oncology, Preclinical Evidence, Clinical Trials, Qualitative Study

## Abstract

**Background:**

The scientific and ethical justification for early phase clinical trials relies on evidence produced in preclinical experiments. Numerous studies have raised concerns about the rigor and reproducibility of such experiments. Little is known about how clinician investigators navigate this contested terrain.

**Methods:**

We conducted semi-structured interviews with 17 clinician investigators involved in early phase neuro-oncology research. Interviews explored their approaches to evaluating supporting evidence during trial development. Data were analyzed using a systematic process based in grounded theory.

**Results:**

Interviews surfaced three themes. First, clinician investigators framed trials as extensions of clinical care. They viewed trials as ways to address the lack of therapeutic alternatives and uphold their role as caregivers. Second, clinician investigators approached review of supporting evidence with an awareness of common evidential vulnerabilities. Assessments relied on heuristics, like the existence of plausible molecular mechanisms or replication. Third, clinician investigators maintained equanimity about trialing in the face of doubts about supporting evidence and unfavorable prospect of therapeutic success. This equanimity was secured by an understanding of themselves as caregivers and by a concern about missing important clinical advances.

**Conclusion:**

Concerns about rigor and reproducibility for preclinical research were understood as tolerable imperfections when trials were supported by plausible claims relating to measured effective sizes and molecular mechanisms. Evidential thresholds for trial initiation were intertwined with clinical objectives of providing care in the face of severe disease. Clinician investigators thus shielded trials from the contestation arising from reproducibility. Ultimately, doubts about preclinical evidence were approached with an equanimity that seldom justified slowing trial efforts.

## Introduction

All major codes of research ethics require that trials show a favorable balance of risk and benefit [1-3]. Early phase trials often pose particular challenges in meeting this condition [4-6]. One reason is that such trials often involve high levels of uncertainty about safety [7-12]. Another reason is that they demand an unusual commitment of time and an unusual research burden from study participants [13-16]. A third reason is that the prospects of benefit, whether in the form of direct benefits to study participants or indirect benefits in the form of knowledge advancement, are at their remotest compared with other modes of clinical investigation [17-20]. Such challenges are especially acute in neuro-oncology, where median survival for patients enrolled in such trials is about a year [21, 22], and where early phase trialing has yielded little in the way of major treatment advances despite decades of vigorous trial activity [23, 24].

Supporting evidence provides a key resource for justifying the risks and burdens of early phase trial enrollment. This evidence takes two basic forms. The first type, toxicology studies, is used to establish the expected tolerability of a treatment regimen, as well as safe starting doses, trial eligibility criteria, and stopping rules for data monitoring [25]. The second type of evidence, preclinical efficacy studies, is used to establish that a clinical hypothesis has sufficient plausibility to redeem the risks and resources needed to test it [4].

Much of the preclinical evidence supporting early phase trials derives from basic science and studies testing investigational treatments in animal models. Over the last two decades, numerous doubts have surfaced about the quality and reliability of such evidence [26, 27]. For example, two major pharmaceutical firms reported being unable to reproduce most basic and preclinical studies performed by academic investigators [28, 29]. In a systematic study of the reproducibility of high impact basic and preclinical studies in cancer biology, fewer than half of studies withstood independent replication; effect sizes in original reports regressed by 80% on average [30]. These findings are complemented by various surveys suggesting concerns about the reproducibility of many preclinical and basic science findings [31]. They are also reinforced by various systematic reviews suggesting that preclinical and basic science studies often do not adequately address internal validity threats (e.g., through methods like blinded outcome assessment or adequate statistical power), construct validity threats (e.g., inappropriate selection of models), or external validity threats (e.g., limited independent replication of original findings) [26, 32-36]. Numerous funders, NGOs, and life science journals have responded to these findings by articulating clearer expectations about rigor and reproducibility [37-41].

The present manuscript is aimed at understanding the way clinician investigators navigate preclinical evidence when planning and conducting early phase trials. We focused our study on neuro-oncology, a field characterized by high research activity, intense clinical demand, and recurrent failure in drug development [24]. We anticipated that high failure rates and the vulnerability of patients with brain cancers might motivate deeper interrogation of supporting evidence during trial development and conduct. To our knowledge, this is the first qualitative study examining how clinician investigators connect their evaluation of supporting evidence to ethical judgment.

## Methods

### Population sample and recruitment

Potential participants were identified by compiling a list of academic and medical institutions across Canada and the United States based on publicly available reports ranking neuro-oncology research programs [42]. From this, we selected clinician investigators based on publicly accessible biographies and co-authorship of neuro-oncology trial publications in the last 9 years. Using purposive sampling, we solicited participants based on their ability to reflect on the intersections between protocol development and clinical care. This required each participant to have both research and clinical obligations. Each participant held, at minimum, a medical degree. We prioritized recruitment of participants who focused their research on primary central nervous system tumors (as opposed to secondary tumors).

This study used in-depth semi-structured interviews about the practices, priorities, and perceptions of neuro-oncology clinician investigators active in early phase trial protocol development. Interviews were designed to uncover how clinician investigators approached the evaluation of supporting evidence in planning early phase trials. Semi-structured interviews were guided by a flexible question template that enabled both consistency among interviews and participant-specific changes to questions. This method was crucial for this study because participants held varying roles during protocol development depending on their specialty and expertise. Each adapted interview guide was thematically organized around the process of protocol development, the evaluation of preclinical evidence, and collaboration (see Supplementary File 1). The interview guide underwent multiple rounds of revisions following discussions between the authors. Following the first interviews, minor changes were made to the guide to focus on pertinent questions and improve interview flow.

Interviews were conducted virtually through Zoom over 7 months (September 2024 to March 2025) and lasted approximately 40 minutes each. Informed consent was obtained prior to the start of each interview. Our study received full and expedited approval from the McGill Research Ethics Board. Demographic information for participants can be found below (Table 1).

### Data analysis

Interviews were processed using Microsoft Word’s built-in transcription software and reviewed manually for accuracy. Transcripts were then analyzed by two coders following a systematic process based on grounded theory. Grounded theory enables the analysis of social practices that underlie the experiences of a population, to inductively build theoretical frameworks [43]. Following this method, we began analysis with the generation of initial codes through a line-by-line, open-coding approach. The ensuing stages of analysis focused on integrating intermediate codes to construct narratives of the social phenomena under study [44-46]. At this stage, the double coding of interview transcripts refined codebook definitions and reduced individual bias. Following grounded theory conventions, we aimed to develop coherent theoretical interpretations that brought together the multiple narratives of participants. To ensure theoretical rigor, numerous discussions took place among the authors, advisory committees, and additional collaborators to sharpen this analytical process and ensure that theoretical interpretations were sound. Quotes were chosen based on their representativeness, typification of code groups, and conciseness. This study was reported using the Consolidated Criteria for Reporting Qualitative Research (COREQ) checklist [47], available as Supplementary File 2.

## Results

We contacted 93 participants across 26 institutions and interviewed 17 participants across 12 institutions. Inductive qualitative analysis gave rise to four pertinent overarching categories, 25 subcategories, and 325 codes (Table 2). Analysis of these categories highlighted three key findings. First, clinician investigators framed trials as extensions of clinical care. This view was buttressed by the acuity of central nervous system tumors, the lack of effective therapeutic alternatives, and an understanding of phase 1 trials as having therapeutic intent. Second, clinician investigators were cognizant of the potential fallibility of preclinical packages. Interviews revealed where participants directed their attention and uncovered common approaches used to generate credibility despite these vulnerabilities. Third, however, clinician investigators exhibited an overall equanimity in their evaluation of preclinical evidence. Concerns about the generalizability of supporting evidence, while present, did not substantially divert clinician investigators from their resolve to initiate trials.

### Trials as extensions of care

Many bioethicists have argued that research and care constitute distinct activities that are bound by different, if overlapping, moral norms [17, 20, 48-53]. Neuro-oncologists, although cognizant of key differences between research and care, viewed the two as entangled. In particular, clinician investigators regarded trials as a progression of care once standard treatments had been exhausted.

I don't find I can separate [clinical trials] from clinical care. I think that there are a lot of people in our field that say every patient with glioblastoma or glioma should be offered a clinical trial at least once or be a candidate for a trial. And in these diseases with such devastating outcomes, the opportunity to engage in something experimental [provides] at least some hope that [their outcome] will be better… (I10, 05:09)

Clinician investigators nevertheless doubted the prospects that patients in early phase trials would benefit directly, or that interventions tested in trials would lead to major advances.

Most clinical trials in neuro-oncology are going to be negative. We know that… (I3, 16:09)

Respondents also acknowledged the risks and burdens presented by participation in early phase trials. Accordingly, clinician investigators emphasized the importance of communicating realistic assessments of risk and benefit to mitigate the ethical tensions between research and care and manage patient expectations.

We're often willing to move forward with something that we perceive to be high risk, given the very poor treatment options available to the patients, with the understanding that any potential participant will be thoroughly informed about the risks and benefits… (I9, 24:36)

Clinician investigators further viewed trials as performing therapeutic functions that were collateral to the pharmacological rationale for trialing.

I think a lot of times [patients] want to have some idea that they're getting something different. Sometimes experimental interventions can have significant risks, but that can also be tied to some hope that the outcome will be different than what's expected for this disease. And so many trials are negative in [neuro-oncology] right. That's one of the big challenges… (I10, 08:15)

Clinician investigators also viewed trialing as a performance of resilience against the reality of neuro-oncology, both for patients and themselves. For patients, trials were seen as a means of fostering a sense of legacy for broader patient communities. For clinician investigators, conducting trials was a means of maintaining morale.

I think in the diseases we're treating, not being involved in trying to find better therapies would be incredibly disheartening because you would constantly just give treatments that have limited efficacy. We need this element of progress, this element of hope, of trying to do a better job. And that's incredibly important for our patients and for the whole brain cancer community. (I12, 14:12)

I do think that [conducting trials] also helps prevent a lot of burnout in providers and clinicians that treat a very devastating disease, knowing that perhaps you could contribute to the bigger picture by showing some of the lessons learned from that [trial]. It gives us a sense of purpose as well. (I6, 16:26)

### Fallibility of preclinical studies

Clinician investigators were mindful of the consequences of running trials based on weak evidence. This was understood in terms of burden for research participants and opportunity cost for research efforts.

…The downstream effect [of initiating trials based on a non-replicated discovery] is that there are then 10 clinical trials trying to ask the same question [based on unreproducible findings]. So, you've now done a lot of work, put a lot of people through a lot of treatment that in retrospect, you would never have done had you known. (I3, 1:12:12)

Many clinician investigators described rules of thumb they used to assess the credibility of preclinical support. A recurrent one was a plausible molecular mechanism of drug action.

The minimum criteria necessary [to justify translation to clinical trials] is to have evidence of a good mechanism and target of action. And evidence that when the drug gets to the target, it can do what we expect it to do. (I12, 26:04)

Counterintuitively, modest effect sizes were often viewed as suggesting greater credibility of supporting evidence.

I like a little bit of failure in that in the model, in the sense that it probably recapitulates the real world better. [For example] when I see something [where] not all the mice survive, but 50% of them did and they seem to be cured. (I13, 07:10)

If you told me that there was reduction of the tumor size by 10%, that is not enough signal to consider this for clinical trial. If you see 100% improvement, obviously this is a very impressive result. But you would have to question whether the authors or the investigators put as much effort as possible into really evaluating this result and validating it. (I5, 25:38)

Many clinician investigators also sought out evidence that the drug could overcome common failure modes in glioma drug development.

…There's a lot of attention paid to whether we know if the agent can cross the blood brain barrier and reach the tumor target sufficiently. [We pay attention to this] if, for example, the preclinical work we're dealing with involves tumor models that were in cell cultures. Or if they were in-vivo xenografts not placed in the central nervous system space… (I9, 17:19)

Independent replication of key studies was also considered a marker of soundness.

[You have to] look at the literature that's available to see if there is a concordance of results from multiple laboratories and different researchers. And that the results are kind of repeatable and consistent across the different platforms… (I1, 24:43)

I think anytime you try to contrive, to control something completely perfectly, you're going to lose somewhere else. And so, because of that, I think the onus is on us to try to make sure we're using rigorous models, multiple different types of models and preferably multiple labs, showing the same thing, not just one group. (I11, 27:38)

Other key practices flagged by clinician investigators related to statistical powering and transparency.

You have to be conscious that preclinical data might not apply to patients because it was done on 10 mice. And then there are so many clinical trials that were launched from preclinical findings that were not effective because they were based on [studies with a] small numbers of assays or samples. So, you need to be cognizant of the limitations… (I8, 18:40)

A lot of the issues [relating to reproducibility] stem from how the actual experiments are reported. … To get [a high] level of precision and [significant] results, it may have been like 10 or 20 tries before they actually got it to work perfectly. I think they have to be more transparent about how many trials they went through, how many times they actually tried where it failed, and when it worked. (I1, 34:56)

### Evidential equanimity

Despite their awareness of preclinical validity threats, clinician investigators did not describe the evaluation of supporting evidence as vexing. Many stated that they typically defer to the judgments of their basic science collaborators.

The background rationale section should be able to write itself. You should be able to work with whoever your basic science colleague is, who's already written that type of background rationale 20 different times, and then you can kind of clean it up in a way that is readable and makes sense. (I13, 20:58)

In general, participants expressed ambivalence or doubt about fastidious demands on supporting evidence. Clinician investigators stressed the importance of adjusting evidence expectations to factors like disease severity.

We're talking about a disease that's uniformly fatal, in months, so we need to accept some gray zone [in the evidential support]. I think that rather than establishing concrete absolute criteria wherein “you've reached X number of studies, you've met the threshold, you can continue,” it's more so a moving target that will involve, probably 3 or 4, if not more, factors that determine what that threshold would look like. And one of them is the safety profile of the intervention, the nature of the disease that we're ultimately treating. (I2, 18:22)

Some clinician investigators had graver doubts about demands on supporting evidence. Reproducibility has been part of the conversation since the beginning of my career in neuro-oncology. But I personally have vacillated how I feel. There are some days where I think, we have nothing [to treat patients with]. I don't care if this [novel intervention] even has a logical mechanism because [example of an approved drug for gliomas] didn’t. (I16, 19:53)

Others saw fastidious evidentiary standards as impediments to their efforts as caregivers and researchers.

…We're often concluding that we shouldn't let the lack of perfect preclinical data interfere with development of anything, if that will lead us to paralysis and not be able to move forward. So, I think we're often considering [reproducibility concerns], but concluding that, … if we're convinced that it's promising, even if imperfect, we'll use [this body of evidence] as rationale for moving forward with the clinical trial. (I9, 27:36)

I think people know that [preclinical] experiments are not always reproducible. So, a lot of potential therapies are just dying in the line before they get translated to the bigger studies because of that skepticism. So, there's two things: we've put forward a lot of drugs that ended up not being effective in the larger trials, but the other side there might have been other things that could have worked in patients that may have not made it through… (I6, 34:42)

Another way investigators qualified their concerns about preclinical evidence was by characterizing non-reproducibility as a normal feature of modeling, not a byproduct of methodological error.

I think the reproducibility issue is not so much a reproducibility issue, but it's understanding that the physiology of mice or rats is not the physiology in humans and that it’s just not a good model. (I1, 34:56)

In this way, clinician investigators protected space for flawed preclinical packages. They viewed known threats to their endeavor, such as statistical underpowering of animal studies and the lack of independent replication, as normal imperfections that should not derail momentum towards trial initiation.

## Discussion

Our qualitative interviews suggest that clinician investigators approach flaws in supporting evidence packages with equanimity. On the one hand, clinician investigators viewed preclinical evidence with caution, acknowledging that it might reflect reporting biases or exaggerated effect estimates. Investigators also doubted that trials would lead to immediate major advances or change the course of disease for participants. In these ways, clinician investigators approached trials as scientific endeavors and embraced the classical Mertonian norm of organized skepticism [54]. On the other hand, this skepticism was tempered by the moral and epistemic merit of clinical trials. Morally, most viewed trials as a vehicle for care and believed the severity of disease sanctioned relaxed standards for initiating trials. Some also worried that too much caution could undermine discovery of effective therapies.

Evidence of a plausible molecular mechanism was generally considered more probative than evidence of a drug’s effect in animal models. To neutralize doubt regarding reporting biases, clinician investigators often used rules of thumb to guide their assessment. These included judgments about the plausibility of a molecular mechanism, and assurance that effect sizes were not overly dramatic. Ultimately, none of our interviewees treated identifying and resolving evidentiary problems as determinative of whether to proceed to a clinical trial.

Our findings align with reports that 20% of oncologists viewed the main societal purpose of clinical trial research as providing state-of-the-art therapy for patient-volunteers [55]. They also align with policy statements, like that of the American Society of Clinical Oncology, that proclaim early phase trials as having “therapeutic intent” [56]. These results are consistent with the findings that early phase trial protocols often provide very limited substantiation of the methods and effects seen in preclinical studies [57]. Building on these findings, our study suggests that clinician investigators evaluate supporting evidence through the lens of a trial’s clinical context.

These findings sit within the larger context of biomedical research that has grappled with preclinical reproducibility. Drug development involves a division of labor between different actors and institutions. These include the pharmaceutical industry, academic medical centers, regulators, journals, public funders, and patient advocates. Over the last decade, many of these actors and institutions have responded to mounting concerns about reproducibility by establishing new policies. Examples include *Nature* statements on improving transparency of reporting and reproducibility [37], the National Academies of Sciences, Engineering, and Medicine consensus report aimed at strengthening research integrity [38], NIH guidance on rigor and reproducibility in grant applications [39], and NIH initiatives towards supporting reproducibility [40].

Our findings help explain a puzzling stance towards reproducibility in biomedical research. We take it as axiomatic that early phase trials should be grounded in a viable clinical hypothesis, and that the viability of a clinical hypothesis rests, to some degree, on the reproducibility and rigor of supporting evidence. Arguably, the stakes of preclinical reproducibility are greatest where interventions are tested for the first time in patients. One might thus anticipate that actors tasked with caring for patients in trials, like clinician investigators, would place particular demands on supporting evidence. While many clinician investigators harbored reservations about the veracity of supporting evidence claims, our interviews suggest clinician investigators were able to make peace with running trials despite these doubts. Clinician investigators proved more concerned about the risk of missing opportunities than about the futility of testing a flawed hypothesis. This representation of risk reflects an understanding that their participation in trials was bound up with their obligations as healthcare providers. In this way, clinician investigators shielded trials from the contested status of the science standing behind them.

Our findings point to several possibilities for strengthening the exchange of perspectives about reproducibility in early phase trial conduct. First, critics of contemporary preclinical research (“reproducibility critics”) are mainly preoccupied with false positives. This reflects the fact that effect sizes in replication studies tend to regress dramatically from those in original studies. However, clinician investigators in realms like neuro-oncology are probably more concerned with false negatives or missed opportunities. Persuading them otherwise would require either showing the rarity with which models fail to detect clinically significant effects or providing a convincing case that false positives are costly to the field. Second, reproducibility critics may be addressing problems that clinician investigators view as orthogonal to their endeavor. Reproducibility critics have directed much of their attention toward preclinical efficacy studies, which test the effect of a drug in an animal model. However, clinician investigators draw heavily on mechanistic evidence. Many mechanistic studies involve different techniques and experimental systems, and to our knowledge, there is little evidence that mechanistic hypotheses undergirding trials are commonly falsified. To persuade clinician investigators they should worry about reproducibility, critics will need to show that mechanistic studies also defy replication, or that mechanistic knowledge, however sound, is grossly insufficient to support trial decision-making. Third, where reproducibility critics tend to view flaws in preclinical studies as roadblocks to clinical investigation, clinician investigators perceive trials as care endeavors. In this way, preclinical evidence seems to corroborate, rather than condition, the decision to launch a trial. Because of their role, clinician investigators may not be the right parties to critically assess rigor and reproducibility. It may be that committees detached from care provision are needed to objectively assess whether a trial should launch. Fourth, we speculate that more resources and attention could be directed towards fostering additional avenues for care beyond trials. Trials are mainly designed and optimized to resolve clinical hypotheses, not to serve a clinical role. Reliance on trials as a means of non-abandonment may reflect the failure of a field to discover or pursue more effective, optimized means of supportive care.

Our findings should be considered in light of several limitations. First, the findings of this study rely on in-depth interviews and thus the recounting of participants’ practices and beliefs. Participant accounts of their own practices may omit important insights that direct observation could have addressed. Second, the sample size of this study also constrains the generalizability of these findings as approaches to protocol development and perceptions of clinical trials may vary across institutional contexts not included in this study. For example, participants were recruited from major academic medical centers that produce high volumes of clinical research. This sampling likely overrepresents institutions with the resources and mandates to develop novel therapeutic interventions. Third, we cannot establish the generalizability of our findings to other clinical areas. Orientations towards preclinical research are likely to have been shaped by the severity of disease and limited effective treatments. Thus, the approaches used by clinician investigators to evaluate evidence in this study may be distinctive to this context.

Fourth, many participants did not specify the sponsorship of their trials. Trials sponsored by pharmaceutical companies tend to demand less input from clinician investigators as supporting evidence may contain proprietary information and study design may be more rigid to fit industry expectations.

## Conclusion

Clinician investigators in neuro-oncology approach the interpretation of supporting evidence in early phase trial protocols with equanimity. They acknowledged the liabilities of preclinical science, including tendencies to produce exaggerated effect sizes. However, they also invoked grounds, epistemic as well as moral, for setting these concerns aside to facilitate trial initiation. Our findings help explain why clinician investigators at the coalface of neuro-oncology trials seem unperturbed by the large and expanding literature raising questions about reproducibility and bias in preclinical science.

## Supplementary Material

This is a list of supplementary files associated with this preprint. Click to download.

- SupplementaryMaterial1SemiStructuredInterviewGuide.docx
- SupplementaryMaterial2COREQGuidelines.docx

## Figures and Tables

**Table 1:** Participant Characteristics (N = 17)

Characteristic	Category		n (%)
**Sex**	Male		12 (71%)
	Female		5 (29%)
**Country**	United States		9 (53%)
	Canada		8 (47%)
**Discipline**	Neuro-Oncology		11 (65%)
	Neurosurgery		4 (24%)
	Radiation-Oncology		1 (6%)
	Hematology-Oncology		1 (6%)
**Professional title**	Professor		7 (41%)
	Associate Professor		4 (24%)
	Assistant Professor		4 (24%)
	Senior Scientist		1 (6%)
	Attending Physician		1 (6%)
**Doctoral Degree**	Yes		9 (53%)
	No		8 (47%)
	Mean	Median	Range
Years of professional experience	22.1	17	8–47
Early-phase trial publications	13.8	5	1–49

**Note.** Institutional affiliations spanned academic medical centers, comprehensive cancer centers, and governmental research institutes across Canada and the U.S.

**Table 2:** Categories and Subcategories Derived from Analysis of Interviews with Clinician Investigators

Categories	Subcategories	Number of Codes
Research vs. Care	Research vs. care in practice	30
	Conflicting obligations	12
	Trials as opportunities	14
	Ethical obligation to trialing	10
	The function of hope	9
Supporting Evidence	Translational divide	14
	Reproducibility	15
	Mediated by acuity	6
	Feasibility	6
	Threshold for “sufficient evidence”	21
	Confidence in models	16
	Preclinical evidence	9
	Translation across disease	8
Protocol Development	Construction of protocols	10
	Concept development	25
	Difficulty during development	24
	Status of inclusion/exclusion	15
	Status of statistical design	6
	Status of safety/tolerance	6
	Status of schedule of events	5
	Status of background/rationale	7
	Status of outcome/end points	11
Ethical Deliberation	Consideration of risk and promise	23
	Therapeutic misconception and overpromise	6
	Impact of trials on patients	7
